# DNA polymerase κ participates in early S-phase DNA replication in human cells

**DOI:** 10.1073/pnas.2405473121

**Published:** 2024-07-01

**Authors:** Feng Tang, Yinan Wang, Ting Zhao, Jun Yuan, Andrew H. Kellum, Yinsheng Wang

**Affiliations:** ^a^Department of Chemistry, University of California Riverside, Riverside, CA 92521-0403; ^b^Environmental Toxicology Graduate Program, University of California Riverside, Riverside, CA 92521-0403

**Keywords:** DNA replication, replication timing, translesion synthesis polymerase, mutagenesis

## Abstract

Cycling cells replicate their DNA during the S phase through a defined temporal program known as replication timing. Mutation frequencies, epigenetic chromatin states, and transcriptional activities are different for genomic regions that are replicated early and late in the S phase. Here, we found from ChIP-Seq analysis that DNA polymerase (Pol) κ is enriched in early-replicating genomic regions in HEK293T cells. In addition, by feeding cells with *N*^ 2^-heptynyl-2′-deoxyguanosine followed by click chemistry–based enrichment and high-throughput sequencing, we observed elevated Pol κ activities in genomic regions that are replicated early in the S phase. On the basis of the established functions of Pol κ in accurate and efficient nucleotide insertion opposite endogenously induced *N*^ 2^-modified dG lesions, our work suggests that active engagement of Pol κ may contribute to diminished mutation rates observed in early-replicating regions of the human genome, including cancer genomes. Together, our work expands the functions of Pol κ and offered a plausible mechanism underlying replication timing–dependent mutation accrual in the human genome.

During the S phase of the cell cycle, DNA replication occurs through a defined temporal program known as replication timing (1). Genomic regions replicated early in the S phase tend to be enriched with clusters of DNase I-hypersensitive sites, exhibit high transcriptional activity, and accrue less mutations than those replicated late in the S phase (1, 2). The latter has also been observed in cancer genomes; however, the detailed molecular mechanisms of how replication timing is regulated and how it modulates mutation rates remain incompletely understood (2).

Translesion synthesis (TLS) is a conserved mechanism for cells to cope with unrepaired DNA lesions by promoting replication across DNA lesions (3). Recent studies also documented noncanonical functions of these polymerases, including DNA repair (4), replicative bypass of secondary structures of DNA, e.g., guanine quadruplex DNA (5), as well as replication of common fragile sites and heterochromatin (6–8). Pol κ is a Y-family TLS polymerase, and it is conserved throughout evolution (3). Pol κ and its *Escherichia coli* ortholog (i.e., Pol IV) have a unique ability to insert efficiently the correct dCMP opposite *N*^ 2^-alkyl-2′-deoxyguanosine (*N*^2^-alkyl-dG) lesions, including those induced by by-products of endogenous metabolism (9, 10). Interestingly, the polymerase is also capable of inserting *N*^ 2^-alkyl-dGTP opposite cytosine in template DNA (9, 11). It remains unclear whether TLS polymerases function differently in early and late S-phase replication and whether such difference contributes to different mutation rates detected in these genomic regions.

## Results

**Fig. 1.**
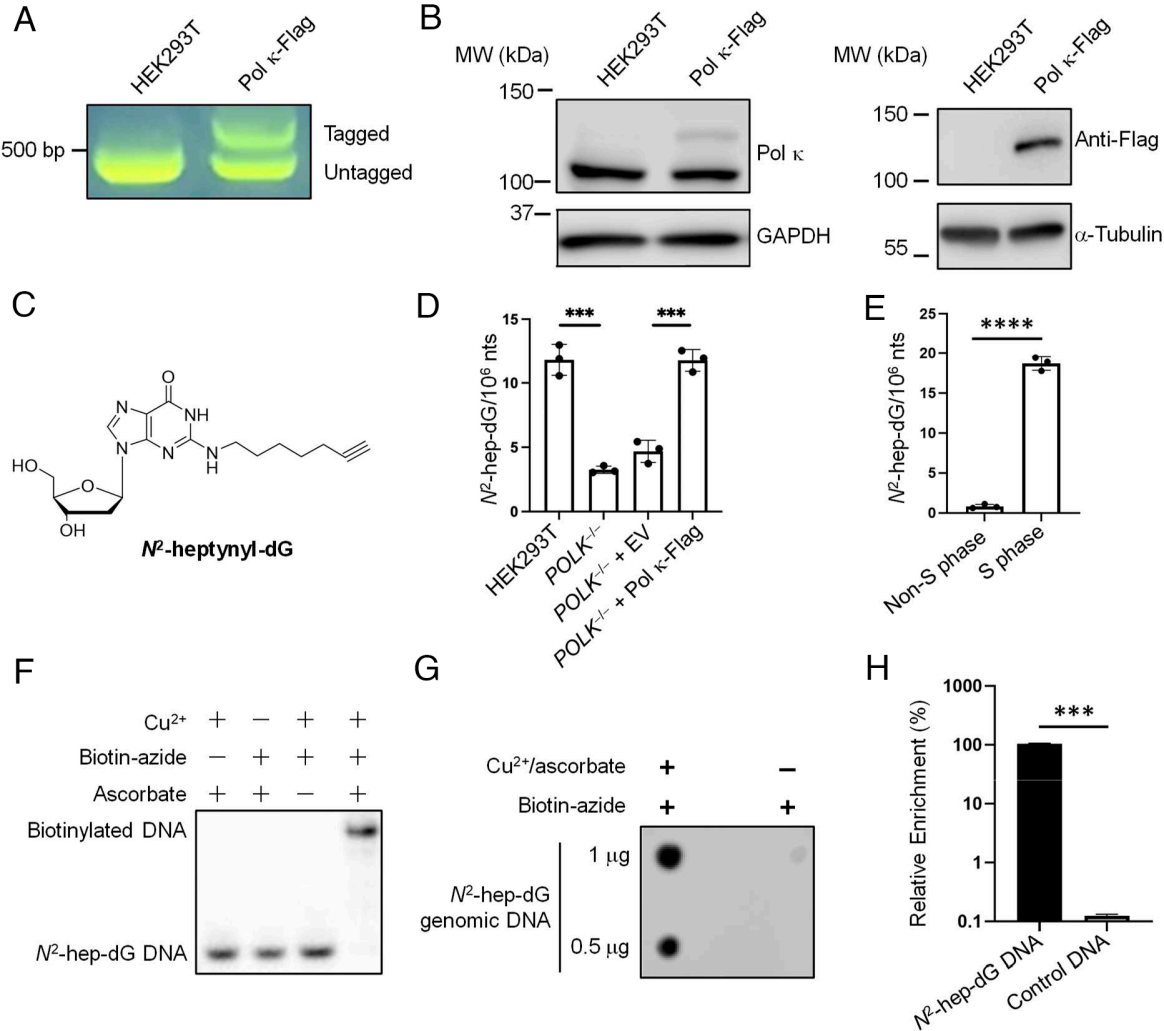
CRISPR knock-in of three tandem repeats of Flag tag to the C terminus of endogenous Pol κ in HEK293T cells and *N*^2^-hep-dG-Seq. (*A*) Agarose gel electrophoresis for detecting insertion of 3×Flag to the C terminus of endogenous Pol κ in HEK293T cells. PCR primers were designed to target outside the homology arms and yield a longer PCR product if the tag is inserted, where the expected amplicon lengths of parental HEK293T cells and the knock-in clone are 424 and 601 bp, respectively. (*B*) Western blot with anti-Flag and Pol κ antibodies for confirming the successful knock-in (KI) of Flag tag to endogenous Pol κ protein, where Pol κ-Flag is expressed at a lower level than the untagged counterpart. (*C*) The chemical structure of *N*^2^-hep-dG. (*D* and *E*) Frequencies of *N*^2^-hep-dG in cellular DNA isolated from asynchronized HEK293T cells, the isogenic *POLK*^-−/−^ cells, and the *POLK*^−/−^ cells transfected with an empty vector or a vector for expressing C-terminally Flag-tagged human Pol κ (*D*), as well as from synchronized S-phase and non-S-phase HEK293T cells (*E*). (*F*) Denaturing gel electrophoresis showing the reaction of a 12-mer oligodeoxynucleotide, d(ATGGCGXGCTAT) (X = *N*^2^-hep-dG), with biotin-azide. (*G*) Dot blot monitoring the conjugation of biotin-azide with genomic DNA isolated from HEK293T cells fed with *N*^2^-hep-dG. (*H*) *N*^2^-hep-dG-Seq enables efficient enrichment of a 90-mer *N*^2^-hep-dG-containing duplex DNA over the corresponding unmodified DNA. The enrichment fold was calculated by normalizing the qPCR-determined recovery rates of *N*^2^-hep-dG-containing DNA over the unmodified counterpart. The data in (*D*), (*E*), and (*H*) represent the mean ± SD of results obtained from three independent experiments. ***P < 0.001; ****P < 0.0001 (unpaired two-tailed Student’s *t* test).

**Fig. 2.**
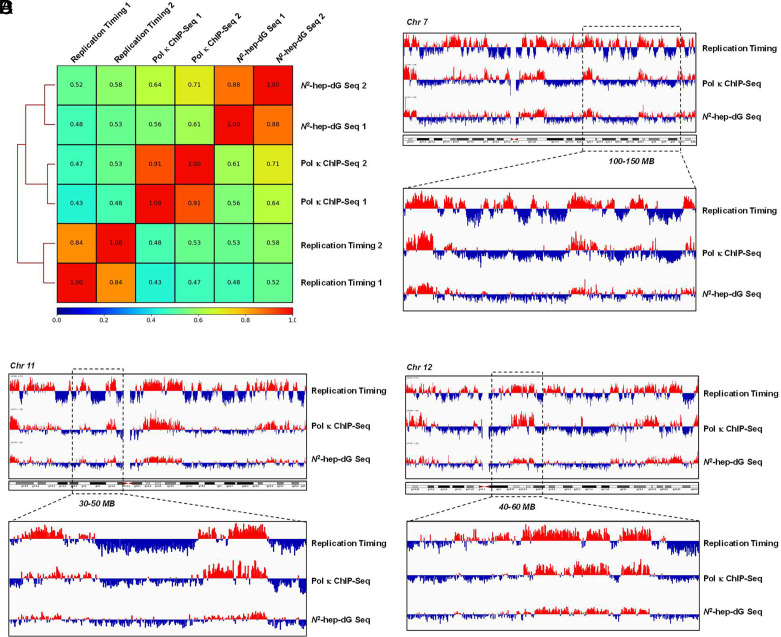
*N*^2^-hep-dG and polymerase κ are enriched at genomic regions replicated in the early S phase of the cell cycle. (*A*) Spearman’s correlation coefficients of replication timing (PRJNA419407), Pol κ ChIP-seq, and *N*^2^-hep-dG-Seq. (*B*) Representative IGV plots illustrating the comparisons of replication timing, Pol κ ChIP-seq, and *N*^2^-hep-dG-Seq results for chromosomes 7, 11, and 12. The bin size for generating the IGV plots in (*B*–*D*) was 50 kb.

On the basis of the unique ability of Pol κ to insert efficiently the correct nucleotide opposite endogenously induced *N*^ 2^-alkyl-dG lesions (9, 10), we hypothesize that this polymerase may function in early S-phase replication, thereby conferring mutation avoidance in these regions of the genome. To test this, we generated CRISPR-engineered HEK293T cells by inserting three tandem repeats of Flag tag to the C terminus of endogenous Pol κ (Fig. 1) and performed ChIP-Seq analysis using anti-Flag antibody. Our results showed that Pol κ is strongly enriched in genomic regions with active early S-phase replication (12), with Spearman’s correlation coefficients being 0.43 to 0.53 from two independent replicates of replication timing sequencing and Pol κ ChIP-Seq results (Fig. 2). In this vein, the results from the two biological replicates of Pol κ ChIP-Seq data are highly consistent, with Spearman’s correlation coefficient being 0.91 (Fig. 2).

We next asked whether Pol κ exhibits polymerase activity in those genomic regions replicated early in the S phase. To this end, we incubated HEK293T cells and the isogenic Pol κ knockout cells (13) with 10 μM *N*^ 2^-heptynyl-dG (*N*^ 2^-hep-dG) for 3 h, isolated genomic DNA from the cells, digested the DNA to single nucleosides, and subjected the nucleoside mixture to LC–MS/MS analysis (14). In this context, we employed *N*^ 2^-hep-dG as a model nucleoside for endogenously induced *N*^ 2^-dG lesions, where the incorporation of an alkyne handle facilitates enrichment of lesion-bearing DNA fragments for next-generation sequencing (NGS) analysis. Our LC–MS/MS results showed that the levels of *N*^ 2^-hep-dG were 11.8 and 3.25 modifications per 10^6^ nucleosides in HEK293T and the isogenic Pol κ knockout cells, respectively (Fig. 1*D*). In addition, reconstitution of Pol κ knockout cells with C-terminally Flag-tagged human Pol κ restored the level of *N*^ 2^-hep-dG incorporation to that detected in parental HEK293T cells (Fig. 1*D*). These results are in line with the previous findings that Pol κ is the major polymerase in incorporating *N*^ 2^-alkyl-dG nucleotides into genomic DNA in human cells (11, 14). The lack of complete abrogation of *N*^ 2^-hep-dG incorporation into genomic DNA in Pol κ knockout cells is likely attributed to a minor role Pol η in incorporating the modified nucleoside into genomic DNA, as observed previously for the structurally related *N*^ 2^-*n*Bu-dG (14). We also examined Pol κ activity in synchronized cells by assessing the levels of *N*^ 2^-hep-dG incorporation, and, in line with the previous observation that the polymerase is translationally up-regulated in the S phase (15), we found that Pol κ’s activity is much higher in S-phase than non-S-phase cells (Fig. 1).

We next employed biotin-azide and click chemistry to enrich *N*^ 2^-hep-dG-containing DNA fragments for NGS analysis (*N*^ 2^-hep-dG-Seq) (Fig. 1). The two replicates of *N*^ 2^-hep-dG-Seq data are again consistent, where the genomic regions with high levels of *N*^ 2^-hep-dG incorporation are also enriched with Pol κ, with the correlation coefficients being 0.56 to 0.71 (Fig. 2). Importantly, overlapping analysis of the *N*^ 2^-hep-dG-Seq and replication timing–Seq data revealed that the sites of *N*^ 2^-hep-dG incorporation are enriched at loci with active early S-phase replication, with Spearman’s correlation coefficients being 0.48 to 0.58 (Fig. 2).

## Discussion

Our Pol κ ChIP-seq results revealed an augmented occupancy of Pol κ in genomic regions that are replicated early in the S phase of the cell cycle, and our *N*^ 2^-hep-dG-Seq results supported the elevated activities of the polymerase in these genomic regions. Considering that *N*^ 2^-alkyl-dG can be induced from by-products of endogenous metabolism and that Pol κ-mediated nucleotide insertion opposite these lesions is both accurate and efficient (9, 10), active engagement of Pol κ in replicating genomic regions early in the S phase may minimize mutation accumulation from endogenously induced DNA lesions. Our findings, together with the documented roles of Pol η and Pol ξ in heterochromatin replication (6, 8), are consistent with the needs of mutation avoidance in actively transcribed genes replicated early in the S phase, while allowing for genome evolution in late-replicating heterochromatin regions (8). Hence, our work expands the functions of Pol κ and offers a plausible mechanism underlying the diminished mutation rates detected in these regions of the cancer genomes (2).

## Materials and Methods

The levels of *N*^2^-hep-dG in genomic DNA were measured using LC–MS/MS. Pol κ ChIP-Seq was conducted using CRISPR-engineered Pol κ-Flag cells and anti-Flag antibody, and *N*^2^-hep-dG-Seq was performed using click chemistry–based enrichment of *N*^2^-hep-dG-harboring DNA fragments followed by NGS analysis. For details, please see online *SI Appendix*.

## Supplementary Material

Appendix 01 (PDF)

## Data Availability

Next-generation sequencing data have been deposited in GEO (GSE220603 and GSE261442) (16, 17). Previously published data were used for this work (SRA with accession number PRJNA419407) (18).
