# Supplementary material for: DNA polymerase κ participates in early S-phase DNA replication in human cells
Source: Proc Natl Acad Sci U S A. 2024 Jul 1;121(28):e2405473121. doi: 10.1073/pnas.2405473121 (PMC11252992; doi:10.1073/pnas.2405473121)
Supplement: Supplementary file 1 — Appendix 01 (PDF) [file pnas.2405473121.sapp.pdf]

**Supplementary Materials for**

# DNA Polymerase κ Participates in Early S-Phase DNA Replication in Human Cells

Feng Tang[1,†], Yinan Wang[1,†], Ting Zhao[2], Jun Yuan[1], Andrew H. Kellum, Jr.[1], and Yinsheng Wang[1,2,*]

[1]Department of Chemistry and [2]Environmental Toxicology Graduate Program, University of California Riverside, Riverside, CA 92521-0403. [†]These authors contributed equally.

*To whom correspondence should be addressed: yinsheng@ucr.edu; Tel: (951)827-2700

## Materials and Methods

### Cell lines

HEK293T cells were purchased from ATCC and tested to be free of mycoplasma contamination using LookOut Mycoplasma PCR Kit (Sigma, MP0035). The cells were maintained in DMEM (Life Technologies) supplemented with 10% FBS (Invitrogen) and 1% penicillin/streptomycin (v/v) at 37°C in a humidified incubator with 5% $CO_2$. For genomic DNA extraction, the cells were harvested at 80% confluency. CRISPR-based integration of three tandem repeats of the Flag epitope tag to the C-terminus of endogenous Pol κ was conducted following the previously reported procedures (1). PCR and Western blot were performed to confirm the correct insertion (Figure 1A-B). The guide RNAs were designed according to a previously described method (2).

### Incorporation of $N^2$-heptynyl-dG ($N^2$-hep-dG) into genomic DNA and $N^2$-hep-dG-sequencing

HEK293T cells and the isogenic *POLK*$^{-/-}$ cells (3) were cultured in a 6-well plate. In reconstitution experiments, the *POLK*$^{-/-}$ cells were transfected with 1 μg of pRK7 plasmid for ectopic expression of C-terminally Flag-tagged human Pol κ or an empty pRK7 vector control, using 3 μL of TransIT-X2® transfection reagent for 24 h. The cells were subsequently incubated for 3 h in culture medium containing 10 μM $N^2$-hep-dG. The cells were then washed twice with 1× PBS, and harvested immediately.

For $N^2$-hep-dG sequencing, genomic DNA was extracted using Monarch® Genomic DNA Purification Kit (New England Biolabs). The isolated DNA was reacted with biotin-azide at room temperature for 6 h (4). The biotinylated genomic DNA was sheared using a Covaris S220 sonicator. The $N^2$-hep-dG-containing DNA fragments were enriched with Dynabeads™ MyOne™ Streptavidin C1 (Thermo). Purified DNA was quantified and verified on an Agilent 2100 Bioanalyzer. The library was then constructed using NEBNext Ultra DNA Library Prep Kit (NEB, E7103S) following the manufacturer's instructions. Subsequently, the purified DNA libraries were assessed using an Agilent 2100 Bioanalyzer and multiplexed for sequencing on a MGISEQ-2000 platform (BGI Genomics). The sequencing reads were checked with FastQC and aligned to the human hg19 reference genome using Bowtie2 with the default configuration (5). Integrative Genomics Viewer (6) was used to visualize the mapping results, and Plotprofile was performed using deepTools at a bin size of 50 kb (7). The sequencing data generated in this study were deposited into the NCBI Gene Expression Omnibus (GEO) under accession number of GSE261442. Replication timing sequencing data were retrieved from Sequence Read Archive (SRA) with accession number of PRJNA419407.

### Western Blot

Approximately 10 μg of whole-cell protein lysate was loaded onto a 8% or 10% SDS-PAGE gel. Antibodies recognizing human Pol κ (Abclonal; 1:2000) and Flag epitope tag (Sigma, 1:2000) were used as primary antibodies for Western blot analysis. Goat anti-rabbit IgG (whole molecule)-peroxidase antibody (Sigma, 1:3000) and m-IgGκ BP-HRP (Santa Cruz Biotechnology, 1:3000) were employed as secondary antibodies. Western blot were also conducted with anti-GAPDH (Santa Cruz Biotechnology, 1:5000) and anti-α-tubulin (Santa Cruz Biotechnology, 1:5000) to confirm equal protein loading.

### Pol κ ChIP-Seq

Chromatin immunoprecipitation of Pol κ was performed as previously described with a few modifications (1). Briefly, $2\times10^7$ cells were cross-linked by adding formaldehyde dropwise directly to the media at a final concentration of 1% (w/v) and rotating gently at room temperature for 10

min. The reaction was quenched by adding 2.5 M glycine to the medium until its final concentration reached 125 mM, and the mixture was incubated at room temperature with shaking for 5 min. After washing with 1× PBS three times, the cells were resuspended in PBS. After centrifugation, the cell pellet was resuspended in RIPA buffer [50 mM Tris-HCl, pH 8.0, 150 mM NaCl, 2 mM EDTA, 1% NP40 (v/v), 0.5% sodium deoxycholate (w/v), 0.1% SDS (w/v) and 1× protease inhibitor cocktail] at 4 °C for 30 min with rotation. Chromatin was sheared using a Covaris S220 sonicator at 4°C for 10 min with a peak incident power of 140, a duty cycle of 10%, and 200 cycles per burst. After centrifugation at 16,000g for 15 min, the supernatant was incubated with pre-blocked anti-Flag M2 affinity gel (Sigma) on a rotator at 4°C overnight. After washing, DNA was eluted from the beads with 120 μL 100 mM $NaHCO_3$ and 1% SDS (w/v) at 68°C for 2 h. Cross-links were subsequently reversed by adding 4.8 μL of 5 M NaCl and 2 μL RNase A (10 mg/mL) and incubated with shaking at 65°C overnight. Proteins in the resulting DNA samples were removed with 2 μL proteinase K (20 mg/mL) and incubated with shaking at 60°C for 2 h. Finally, the DNA was purified using Cycle Pure Kit (Omega). Purified DNA was quantified and verified on an Agilent 2100 Bioanalyzer. The library was then constructed using NEBNext Ultra DNA Library Prep Kit (NEB, E7103S) following the manufacturer's instructions. Subsequently, the purified DNA libraries were assessed using an Agilent 2100 Bioanalyzer and multiplexed for sequencing on an MGISEQ-2000 platform (BGI Genomics).

The sequencing reads of ChIP-seq were checked with FastQC and aligned to the human hg19 reference genome using Bowtie2 in the default configuration (5). After alignment, the sequencing duplicates were removed using Picard MarkDuplicates (http://broadinstitute.github.io/picard/). Integrative Genomics Viewer (6) was used to visualize the mapping results. The ChIP-Seq data generated in this study have been deposited into the NCBI Gene Expression Omnibus (GEO) under accession number GSE220603.

**LC-MS/MS**

LC-MS/MS measurement of the incorporation of $N^2$-hep-dG was conducted following previously published procedures (8). Briefly, genomic DNA was extracted from cells using Qiagen DNeasy Blood & Tissue Kit following the manufacturer's instructions. Extracted genomic DNA was subjected to enzymatic digestion. In brief, 1 μg of cellular DNA was digested with 2.5 units of benzonase (Sigma), 0.003 unit of phosphodiesterase I (Sigma) and 2 unit of alkaline phosphatase (Sigma) in a 50-μL buffer containing 20 mM Tris-HCl, pH 7.5, 100 mM NaCl, 20 mM $MgCl_2$ at 37°C for 18 h. The enzymes in the digestion mixture were then removed by chloroform extraction. The aqueous phase was dried *in vacuo* and the resulting residues redissolved in 10% methanol in $H_2O$ for LC-MS/MS analysis.

The LC-MS/MS experiments were conducted on an Q Exactive Plus quadrupole-Orbitrap mass spectrometer equipped with a Nanospray Flex source and coupled with a Dionex UltiMate 3000 RSLCnano UPLC system (Thermo Fisher Scientific, San Jose, CA, USA). The nucleoside mixture was separated using an in-house packed trapping column (150 μm × 40 mm) and an analytical column (75 μm × 200 mm), packed with Zorbax SB-C18 (5 μm in particle size, 200 Å in pore size, Michrom BioResource, Auburn, CA). Formic acid (0.1%, v/v) in water and formic acid (0.1%, v/v) in 80% acetonitrile were used as mobile phases A and B, respectively. A gradient of 1-95% B in 20 min, and 95% B for 20 min was employed, where the flow rate was 300 nL/min. The mass spectrometer was operated in the positive-ion mode with an electrospray voltage of 1.5 kV, and the capillary temperature was 250°C. The precursor ions were fragmented in an HCD collision cell at a normalized collision energy of 20. The MS/MS resolution was 35,000, the automated gain control target was $1 \times 10^6$, and the maximum accumulation time was 50 ms. MS/MS for the $[M + H]^+$ ions of $N^2$-hep-dG (*m/z* 362.2) and [$D_9$]-$N^2$-*n*Bu-dG (*m/z* 333.2) was monitored. [$D_9$]-$N^2$-*n*Bu-dG was used as a surrogate standard for quantifying $N^2$-hep-dG, and a

calibration curve was constructed by mixing *N*$^2$-hep-dG and [$D_9$]-*N*$^2$-*n*Bu-dG at defined molar ratios and analyzing the mixtures with LC-MS/MS under the same experimental conditions.

**Cell synchronization and *N*$^2$-hep-dG incorporation**

HEK293T cells were synchronized by following a previously described double thymidine block protocol with minor modifications (9). The cells were plated at 20% confluency in a T75 culture flask containing 10 mL of DMEM supplemented with 10% fetal bovine serum (FBS) and 1% penicillin/streptomycin. Two hundred µL of 100 mM thymidine was added into the culture media until its final concentration reached 2 mM. The cells were incubated for 14 h and then washed with 10 mL pre-warmed PBS twice. The cells were subsequently incubated with 10 mL pre-warmed DMEM supplemented with 24 µM 2'-deoxycytidine for 9 h. Two hundred µL of 100 mM thymidine was added to the culture media again and the cells were incubated for 14 h. The cells were washed twice with 10 mL pre-warmed PBS and incubated in the pre-warmed DMEM supplemented with 24 µM 2'-deoxycytidine and 10 µM *N*$^2$-hep-dG for 3 hrs, which is considered as S phase cells. The non-S phase cells were treated with 10 µM *N*$^2$-hep-dG for 3 hrs after double thymidine block without releasing.

## References


1. F. Tang, Y. Wang, Z. Gao, S. Guo, Y. Wang, Polymerase η recruits DHX9 helicase to promote replication across guanine quadruplex structures. *J. Am. Chem. Soc.* **144**, 14016-14020 (2022).
2. F. A. Ran, P. D. Hsu, J. Wright *et al.*, Genome engineering using the CRISPR-Cas9 system. *Nat. Protoc.* **8**, 2281-2308 (2013).
3. J. Wu, L. Li, P. Wang *et al.*, Translesion synthesis of $O^4$-alkylthymidine lesions in human cells. *Nucleic Acids Res.* **44**, 9256-9265 (2016).
4. S. I. Presolski, V. P. Hong, M. G. Finn, Copper-catalyzed azide-alkyne click chemistry for bioconjugation. *Curr. Protoc. Chem. Biol.* **3**, 153-162 (2011).
5. B. Langmead, S. L. Salzberg, Fast gapped-read alignment with Bowtie 2. *Nat. Methods* **9**, 357-359 (2012).
6. J. W. Nicol, G. A. Helt, S. G. Blanchard, Jr., A. Raja, A. E. Loraine, The Integrated Genome Browser: free software for distribution and exploration of genome-scale datasets. *Bioinformatics* **25**, 2730-2731 (2009).
7. F. Ramirez, D. P. Ryan, B. Gruning *et al.*, deepTools2: a next generation web server for deep-sequencing data analysis. *Nucleic Acids Res.* **44**, W160-165 (2016).
8. Y. Tan, S. Guo, J. Wu *et al.*, DNA Polymerase h promotes the transcriptional bypass of $N^2$-alkyl-2'-deoxyguanosine adducts in human cells. *J. Am. Chem. Soc.* **143**, 16197-16205 (2021).
9. H. T. Ma, R. Y. Poon, Synchronization of HeLa cells. *Methods Mol. Biol.* **761**, 151-161 (2011).
